# Fecal microbiomes from screening sampling tubes are stable despite varying sampling and storage conditions

**DOI:** 10.1038/s41598-025-12506-5

**Published:** 2025-07-24

**Authors:** Anders Bech Jørgensen, Louise Almer, Birgitte Brandstrup, Lennart Friis-Hansen

**Affiliations:** 1 Department of Surgery, Part of Copenhagen University Hospitals – Holbaek, Smedelundsgade 60, Holbaek, 4300 Denmark; 2 Center for Translational Research, Copenhagen University Hospital – Bispebjerg and Frederiksberg, Nielsine Nielsensvej 4B, Copenhagen NV, 2400 Denmark; 3 Department of Clinical Biochemistry, Copenhagen University Hospital – Bispebjerg and Frederiksberg, Nielsine Nielsensvej 4B, Copenhagen NV, 2400 Denmark

**Keywords:** Fecal Microbiome, 16S rRNA, Full-length, Oxford Nanopore Technologies, Sampling, Storage, Microbiome, Translational research, Next-generation sequencing, Immunohistochemistry

## Abstract

Residual material from fecal immunochemical test (FIT) tubes, commonly used in colorectal cancer screening programs, offers a valuable resource for large-scale gut microbiome studies. With recent advances in sequencing technologies, sequencing the full-length bacterial 16S ribosomal gene is now feasible. In this study, we evaluated the impact of pre-analytical handling conditions on microbiome profiling using FIT samples. Stool samples from eight healthy adults were subjected to various short-term (+ 20 °C) and long-term (–18 °C or –80 °C) storage conditions prior to DNA extraction. We also investigated the effects of sampling variation and the presence of buffer medium. Full-length 16S rRNA gene amplicons were generated and sequenced using Oxford Nanopore Technology to characterize the microbiome composition. Despite variations in sampling and storage conditions, microbiome richness, Shannon diversity, and individual characteristics were preserved, demonstrating the robustness of microbiomes extracted from FIT tubes. However, some variations were noted, such as increased amounts of collagenase-producing bacteria from 0.2 to 0.6% to 1.7–2.6% in samples stored at +20 °C for 4–10 days. Despite unsupervised and varying sampling and storage conditions, the fecal 16S rRNA microbiomes remained representative and robust. These findings support the usability of FIT samples for large-scale population microbiome research.

**Supplementary Information:**

The online version contains supplementary material available at 10.1038/s41598-025-12506-5.

## Introduction

Genetic and environmental factors contribute to the significant interindividual variability in human susceptibility to disease. The gut microbiota composition and associated metabolites are influenced by several environmental factors that have garnered increasing attention due to mounting evidence of their substantial impact on host health, disease susceptibility, and recovery^1^. However, the gut microbiota and host health relationship is complex and bidirectional, as they coevolve and mutually adapt^2^. The host’s diet, exercise, immune status, chronic illnesses, and other factors (such as age, antibiotics, and environmental conditions) affect gut microbiota composition and metabolite production^3^.

Analyses of the gut microbiome are becoming “standard tests” used in basic science, population studies, and daily clinical practice^4^. However, the lack of common standards has made reproducing results from other laboratories challenging for one laboratory^5^. The preanalytical handling of samples is now a well-recognized source of considerable variation in daily clinical laboratory tests^6^ and microbiome analysis^7^. Therefore, guidelines have recently been developed to help standardize preanalytical procedures and workflows, including collection, transport, and storage before analysis, to facilitate the harmonization and commutability of test results through reduced interlaboratory variation^8^. In clinical practice and research, gut microbiome analyses often require grams of feces to be collected in a transport device and shipped to the laboratory, where the stool sample is preferably homogenized before an aliquot is analyzed^9^. However, although the collection is noninvasive and practical, the implementation and adherence to stool analyses have been hindered by multiple challenges, including forgetfulness and avoidance^10^ and difficulties with the stool collection itself^11^.

Fecal immunochemical test (FIT) based colorectal cancer screening programs also suffer from many of the above challenges, although most programs have a 60–70% uptake rate^12^. Moreover, several studies have demonstrated the feasibility of extracting sufficient bacterial DNA from residual buffer solutions of 1.5 mL or more for subsequent sequencing of gut microbiomes^13–19^. Therefore, FIT sampling tubes could be an inexpensive and easy way to conduct population-based microbiome studies and may also improve the program’s performance and predict, for instance, the risk of complications to surgery. However, unlike “the usual” collection methods for clinical and research microbiome testing, the collection of FIT samples differs in several ways^20^. First, the samples were not intended for microbiome analysis, as only approximately 10 mg of feces was collected. The participants received only brief written instructions emphasizing the importance of collecting samples from the stool surface and returning them by mail. Neither the time of collection nor the duration of the mail transport is recorded, nor is the temperature at which this is done known. Since these conditions are given when using regular mail services, the focus of the usual verification process had to be turned “upside down.” Instead, the focus had to be on the impact of these factors on sample stability and, consequently, how representative the FIT samples are of the microbiome in the patients.

Several studies have shown that FIT sample DNA seems stable at room temperature in collection buffer^13–19^ when 0.5 kilobase (kB) PCRs are performed for microbiome analyses by amplifying the V3–4 regions of the 16S ribosomal RNA (rRNA) gene. The emergence of Oxford Nanopore Technologies (ONT) and other long-read sequencing methods allows routine sequencing of the entire V1–V9 region of the 16S rRNA gene in one read. However, the average size of the DNA fragments isolated from the FIT samples is approximately 0.9 kB^17^ which is shorter than the 1.6 kB V1–V9 region, resulting in a lower yield of DNA than when the 0.5 kilobase V3–V4 region is sequenced^21–23^. In “routine” clinical and research settings, this is usually compensated for by increasing the amount of DNA extracted.

Therefore, we examined the impact of preanalytical handling of FIT samples (sampling from the surface versus core of the stool, short- and long-term storage at different temperatures, and the impact of the transport medium) on the performance of bacterial 16S rRNA sequencing via ONT. To further investigate the effects of possible preanalytical variation on the biological interpretation of the test results, we, as an example, examined the variation in the detection of bacteria with the potential to produce the enzyme collagenase.

## Results

We included eight adult volunteers (five women and three men) in the study, with a median age of 39 years (28–53).

### Quality control and sequencing depth

**Fig. 1:**
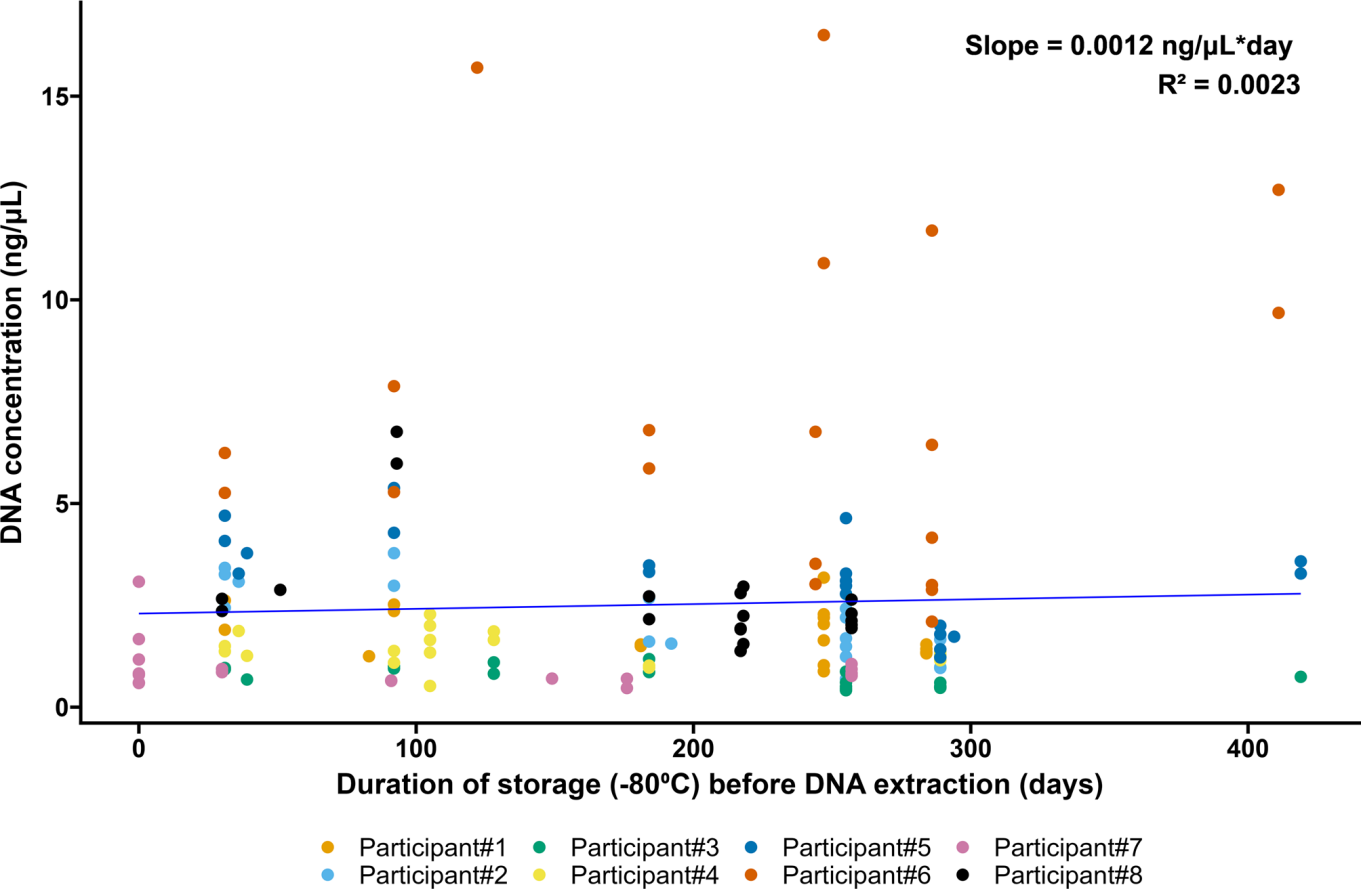
A scatter plot of the DNA concentration following extraction as a function of storage duration at −80 °C before the bacterial DNA was extracted. The straight line is a Deming regression line.

Bacterial DNA was extracted, and microbiomes were sequenced from all samples. One sample was lost during processing (the transport medium sample of Participant #2). The read length of the DNA strands was 1,436 base pairs (1,432–1,440 base pairs), and the quality (Phred) score was 13.3 (13.0–13.7). The DNA concentration in the sampling tubes remained unchanged when stored at −80 °C for up to 400 days (Fig. 1).

**Fig. 2:**
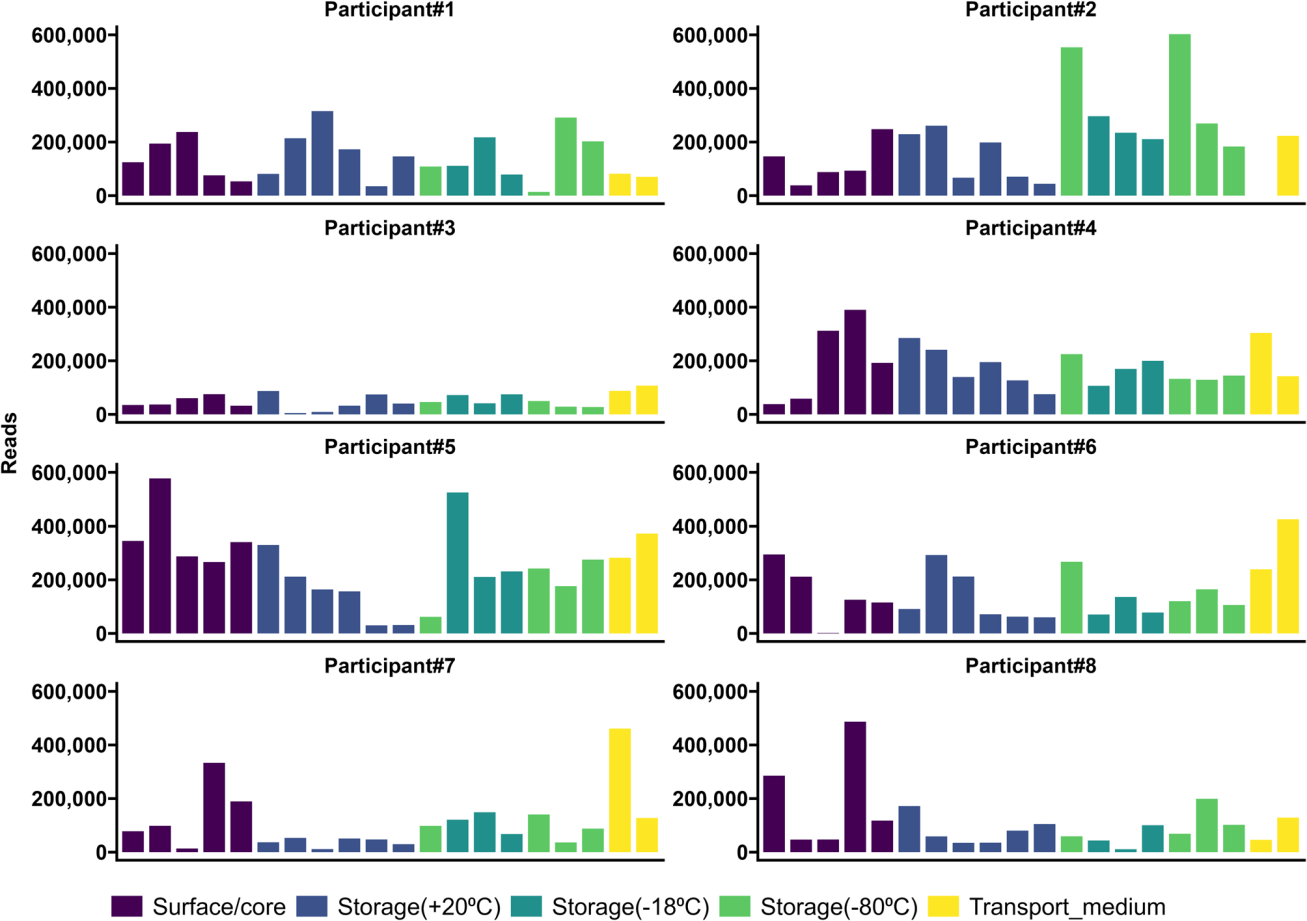
Bar plot of the number of reads in all samples, shown for each participant and divided into the five subgroups (surface vs. core, short-term storage (+ 20 °C), long-term storage (–18 °C or –80 °C), or buffer vs. water). One sample from Participant #2 was lost, hence the zero read count. The scale on the y-axis is fixed in all plots to make comparing the participants easier.

**Fig. 3:**
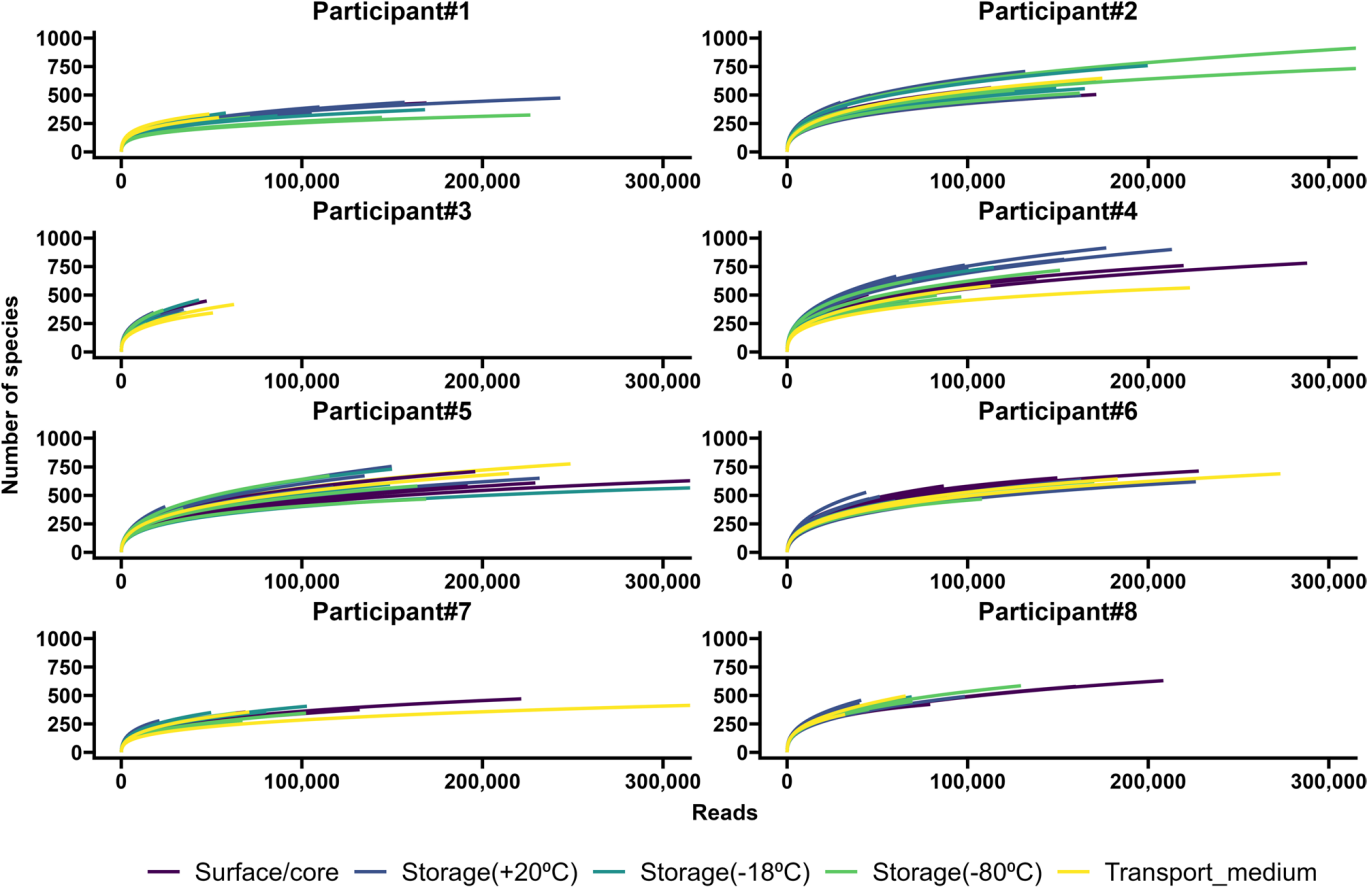
Rarefaction curves of all participants. The five subgroups (surface vs. core, short-term storage (+20 °C), long-term storage (–18 °C or –80 °C), or buffer vs. water) are colored separately (colors similar to the corresponding bars in Fig. 2). The scale on the y-axis is fixed in all plots to make comparing the participants easier.

**Fig. 4:**
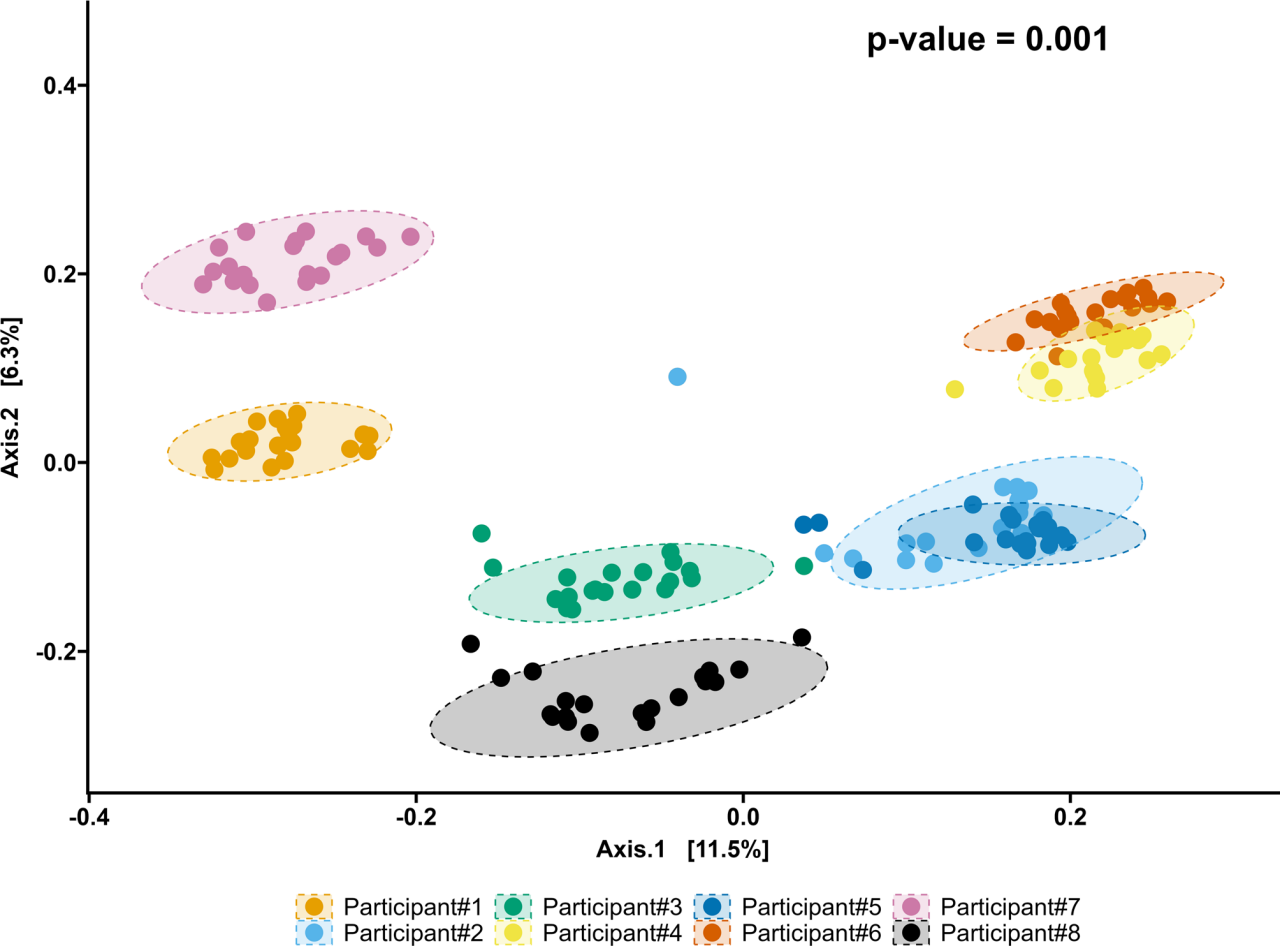
Principal Coordinates Analysis plot (Canberra distance) of all samples. The Axis.1 depicts the dimension with the most significant variation (in percent) of the samples and the Axis.2 depicts the dimension of the second-most variation (in percent). Differences in beta diversity between the participants were calculated using the permutational analysis of variance (PERMANOVA) test.

The median number of reads for all the samples was 116,691 (range 1,956–602,613). Participant #3 had the lowest number of reads (43,644; range 4,943–107,291), and Participant #5 had the highest (254,414; range 30,062–578,435). Most samples (*N* = 143) had fewer than 300,000 reads. The accuracy of the Basic Local Alignment Search Tool (BLAST) alignment was 94.6% (91.1–96.4%). The samples with the most reads appeared to be randomly distributed across different sequencing runs (Fig. 2). The rarefaction curves illustrate the sequencing depth of each sample (Fig. 3). The PCoA plot revealed that all participants clustered differently (Fig. 4).

### Preanalytical variation

#### Sampling site

No significant variation in species richness, alpha diversity, or relative abundance was detected between surface and core fecal samples (Supplementary Table S1 and Supplementary Fig. S1a-b).

#### Short-term storage (+20 °C)

We found no differences in richness or alpha diversity (Supplementary Table S1). The relative abundance visualization revealed minor changes at the phylum and genus levels during the ten days of storage at room temperature, although none were significant (Supplementary Fig. S2a-b).

#### Long-term storage (–18 °C or –80 °C)

There were no significant differences in richness or alpha diversity when the samples were stored at −18 °C or −80 °C (Supplementary Table S1). The relative abundance visualizations revealed that several phyla and genera presented minor variations among the samples stored for 1, 3, and 6 months, but none of the changes were significant (Supplementary Fig. S3a-b).

### Impact of the transport medium

We found no difference in richness or alpha diversity between the buffer and water samples (Supplementary Table S1). The visualization of the relative abundance could indicate slight changes in the *Bacillota*/*Bacteroidota* ratio and the amounts of *Blautia* and a few other genera (Supplementary Fig. S4a-b). However, the relative abundance calculations revealed no differences between the groups.

In summary, the PCoA plot revealed that although there were minor differences within each participant’s samples, the most significant differences were observed between different individuals. Moreover, the PCoA confirmed that the intraindividual variation in the fecal microbiome between different sampling and storage methods was insignificant; thus, interindividual variations were preserved in the analyses.

### Bacteria with the potential to produce collagenases

**Fig. 5:**
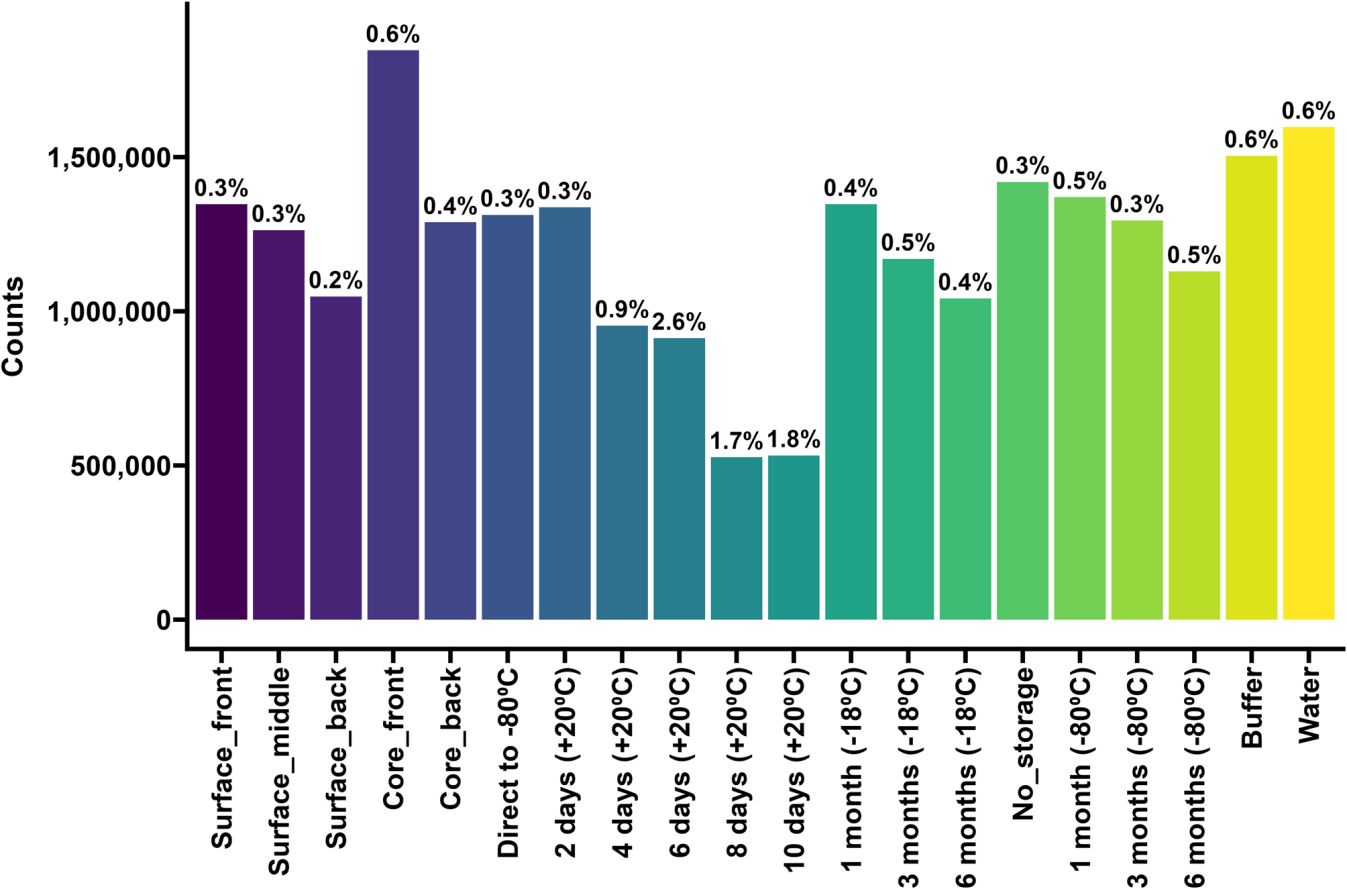
Bar plot of all counts, divided into categories of sample type. The prevalence of bacterial species that could produce collagenase is displayed at each bar.

**Fig. 6:**
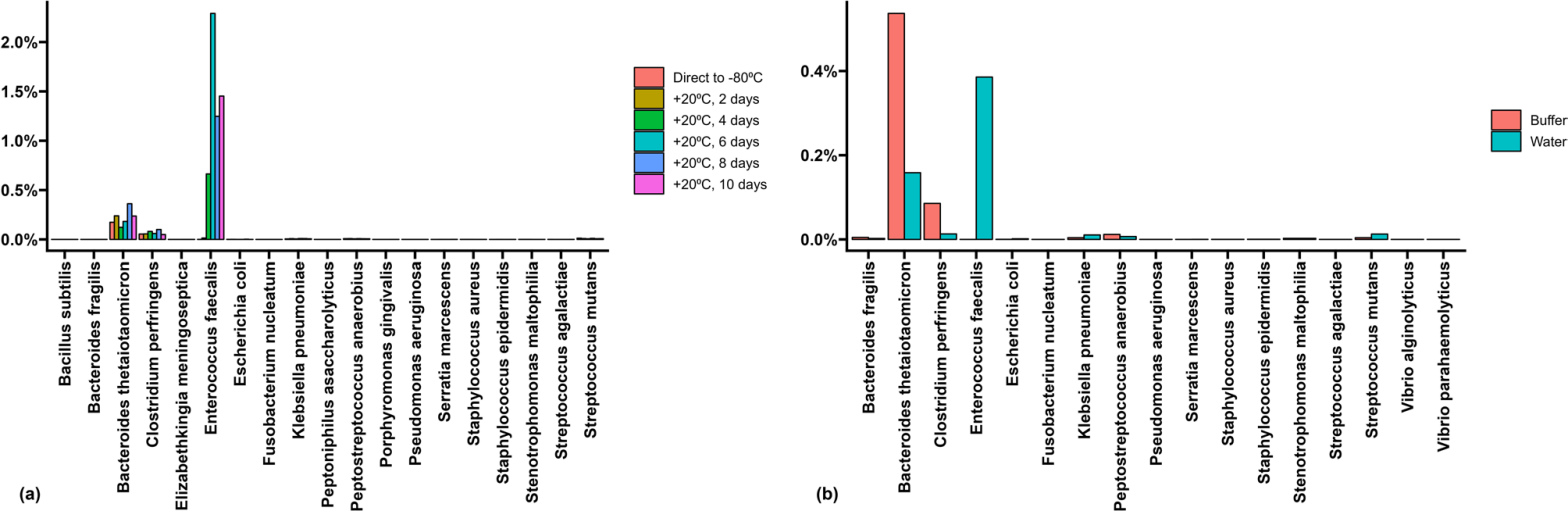
Bar plots of the prevalence of the bacterial species with the potential to produce collagenase among **(a)** Short-term storage (+20 °C) samples, and **(b)** Transport medium (buffer vs. water) samples.

The subgroup of bacteria with the potential to produce collagenase (according to a recent study^24^) constituted 0.2–0.6% of most samples containing the FIT buffer medium (Fig. 5). However, their abundance increased to 1.7–2.6% after the samples were stored for four days or more at +20 °C. In contrast, *Bacteroides thetaiotaomicron* and *Clostridium perfringens* constituted approximately the same proportion in all the short-term storage samples. The fraction made up of *Enterococcus faecalis* increased over time (Fig. 6a). *Enterococcus faecalis* was present in samples where the buffer medium had been replaced with sterile water, but was absent in the FIT buffer medium samples (Fig. 6b). In the surface vs. core samples or long-term storage samples, the amount of *Enterococcus faecalis* was minor and did not seem to vary (Supplementary Fig. S5–S6).

## Discussion

This study revealed that enough 1.5 kb of bacterial DNA could be extracted from the FIT sampling tubes to amplify the entire bacterial 16S rRNA region for microbiome analysis. We also found that the characteristics of the individual microbiomes derived from the +1.5 mL residual material of the FIT sampling tubes were stable despite various sampling, transport, and storage conditions. This parallels what has been observed for V3–V4-based microbiomes derived from residual material from FIT sampling bottles^13–19^. However, the fraction of bacteria that have the potential to produce collagenase increased with the duration of short-term storage at room temperature in the FIT buffer medium and in the sampling tubes with sterile water, primarily due to the growth of *Enterococcus faecalis*.

Our analysis revealed that alpha diversity was comparable between surface and core samples across various stool segments, corroborating the findings of an earlier study^25^. In contrast, we did not find significant differences in the relative abundance compositions of several bacterial families. Another study has shown that replicating the detection of low-abundance taxa in samples taken 1 cm apart in each stool was challenging^26^ indicating that localized sampling only partially represents the fecal microbiome. Furthermore, stool consistency is correlated with species richness, with liquefied stool having the lowest richness^27^. Unfortunately, we did not collect information about stool consistency in our study.

There has yet to be a consensus on how to collect, store, sequence, and process data from gut microbiome studies^28–31^ nor which FIT sampling tube to use^32,33^. We detected minimal variation in the gut microbiome collected from different parts of the feces when the OC–Sensor sampling system was used. This contrasts with other reports suggesting that samples taken from similar locations should be homogenized before further processing to prevent bias from sampling variation^34^. The microbiome composition remained unaffected when comparing aerobe and anaerobe sampling tubes^35^. Previous research has examined the impact of varying storage durations and temperatures, revealing that storage at room temperature for up to 48 hours^25,36,37^ or even 7–14 days^38,39^ did not change the microbiome composition. In contrast, variations were detected as soon as 15 min in other studies^40,41^. We showed the microbiome was stable after short-term storage at a refrigerator temperature (+4 °C) for up to 48 hours^37^. After storage at −18 °C (like a household freezer), the microbiome composition was stable for at least three days^40^. The gold standard for sample storage is −80°C^42^, and recent studies revealed that the microbiome composition was stable at this temperature after 12 months of storage^43,44^. In the absence of −80 °C facilities, freeze-drying is a suitable alternative^45^. Keeping the storage temperature constant is essential to avoid freeze-thawing of the samples, thus damaging the DNA^46^. Nevertheless, DNA and RNA levels were stable even after nine freeze-thaw cycles in a study of colorectal cancer tissue^47^. The buffer medium in the sampling tube may allow for postsampling growth of different bacteria, thereby altering the composition of the gut microbiome before DNA extraction. A wide range of buffers is used in microbiome studies. Overall, the composition of the bacteria was stable in these buffers, although variations in bacterial taxonomy can occur depending on the buffer used^48–51^. Most importantly, we found that the characteristics of each individual’s gut microbiome were captured irrespective of storage conditions, as the variations originating from differences in storage conditions were minor compared with the interindividual microbiome variation of the donors, which aligns with other studies^52^.

The HEPES buffer medium of the sampling tubes is a widely used buffering agent in biological and biochemical research and helps maintain a stable pH within the physiological range. We found that the growth of *E. faecalis* was initially limited by the HEPES buffer compared with sterile water. Nevertheless, storage at room temperature allowed bacterial growth in samples containing buffer medium and stored for 4–10 days. Other types of transport media (Amies and Cary–Blair) resulted in similar growth of *E. faecalis* after 24–48 h at room temperature, but storing the samples at +4 °C reduced growth^53,54^.

Using the residual buffer material from FIT screening in an OC–Sensor, and possibly other FIT sampling tubes, allows for the accessible collection and analysis of baseline microbiomes, which reflect the adaptation of the microbiomes to the host and vice versa. They, therefore, reveal characteristics of both the gut microbiome and the host. We also found that the standard preanalytical handling of the FIT samples (collection and subsequent transport by mail) had a minimal effect on the composition of the samples, ensuring that the individual characteristics of the microbiomes were preserved. Finally, we demonstrated that microbiomes can be generated from DNA extracted from residual material in FIT sampling tubes, based on the sequence of the full-length V1–V9 hypervariable regions of the 16S rRNA gene. Using the entire V1–V9 sequence, rather than shorter areas, such as the V3–V4 region, has enabled more accurate discrimination between closely related species^21^.

A strength of this study is that we addressed possible concerns regarding the impact of variations in the preanalytical handling of the types of sampling tubes used in the Danish Colorectal Cancer Screening Program. The results for the fecal microbiomes originating from these types of samples represent the species present, regardless of variations in sampling techniques or storage conditions. A limitation of the study is that we did not use positive controls. During the DNA extraction and PCR steps, we analyzed samples containing sterile water as the sample material, which constitutes negative controls. The results should be interpreted cautiously, especially concerning low-abundance bacteria, as both localized sampling and the choice of primer could impact these findings. Another limitation is the duration of “long-term storage” (6 months). The preanalytical stability of samples stored for several years remains unknown. This study supports the feasibility and reliability of large-scale studies of fecal microbiomes collected from the sampling tubes of a colorectal cancer screening program. Nevertheless, the limitations of microbiome studies should always be taken into account when designing future studies.

We have demonstrated that bacterial DNA can be successfully extracted from OC–Sensor FIT screening sampling tubes and subsequently used to sequence full-length 16S rRNA microbiomes. Furthermore, we found that the collection site (superficial vs. core and front, middle, or tail) of the fecal matter did not significantly impact the composition of the fecal microbiome, nor did the composition change due to short-term (a surrogate for transport time) or long-term storage at various temperatures. Furthermore, despite the limited growth of robust bacteria (e.g., *Enterococcus faecalis*), the sampling buffer stabilized the bacteria, allowing for minimal growth compared to sterile water. Therefore, the full-length 16S rRNA microbiomes generated from the residual FIT sampling material collected through FIT-based CRC screening programs are representative of the feces at the time of collection. This study lays the groundwork for microbiome research by utilizing sampling tubes from colorectal cancer screening programs.

## Materials and methods

This paper was written according to the ”Strengthening The Organization and Reporting of Microbiome Studies” (STORMS) checklist^55^ (Supplementary Material). The study received ethical approval from the Committee of Research Ethics in Region Zealand (SJ-1069).

### Study participants

This study was an observational cohort study. Healthy adult volunteers provided stool samples following specific sampling instructions. None of the participants took daily medication that could modify the gut microbiome composition (e.g., proton pump inhibitors, nonsteroidal anti-inflammatory drugs, opioids, antibiotics, or chemotherapy) within three months of sampling.

### Sample collection

Sampling was performed at the participants’ homes, following written instructions from the Danish Colorectal Cancer Screening Program (DCCSP), which included a cartoon illustrating how to collect the fecal samples. The fecal matter was gathered via the EasySampler Basic paper toilet cover (GP Medical Devices, Holstebro, Denmark). Afterward, samples were collected using an OC–Sensor (OC–Auto Sampling Bottle 3; Eiken Chemical Co., Ltd., Tokyo, Japan) sampling tube. The OC–Sensor is designed to collect fecal matter for a fecal immunochemical test and features a flattened tube with a cap attached to the sampling probe^56^. The bottom of the sampling tube features a foil seal, allowing access to the sample buffer in which the fecal matter is dissolved. The yield is approximately 10 mg of feces per sample, corresponding to a feces concentration of 5 mg/mL. The transport medium in the sampling tubes was 2 ml of HEPES buffer (N-2-hydroxyethylpiperazine-N’−2-ethane-sulforic acid). When used in the DCCSP, only a few microliters of buffer are used to analyze for the presence of hemoglobin. The remaining buffer medium, therefore, corresponds to the residual material mentioned in the Introduction. For each participant, 20 stool samples were collected from one defecation. The time from sampling in the participants’ homes until the samples were processed was planned to last 1–3 days to mimic the anticipated time for the sample from the patient participating in the DCCSP to laboratory processing in a real-life setting. During this period, the participants were instructed to keep the samples at room temperature to mimic transport by regular mail.

### Sample handling

The participants were instructed to sample superficially from the stool’s proximal, middle, and distal parts to investigate the differences in bacterial distribution throughout the feces. Additionally, two samples from the core of the stool were collected by introducing the tip of the sampling device into the center of the stool at two locations and then twisting it a couple of times. To mimic the transport conditions for patient samples before they reach the laboratory performing the FIT, six localized superficial samples were pooled and stored at room temperature for up to 10 days before being stored at −80 °C until further processing.

**Fig. 7:**
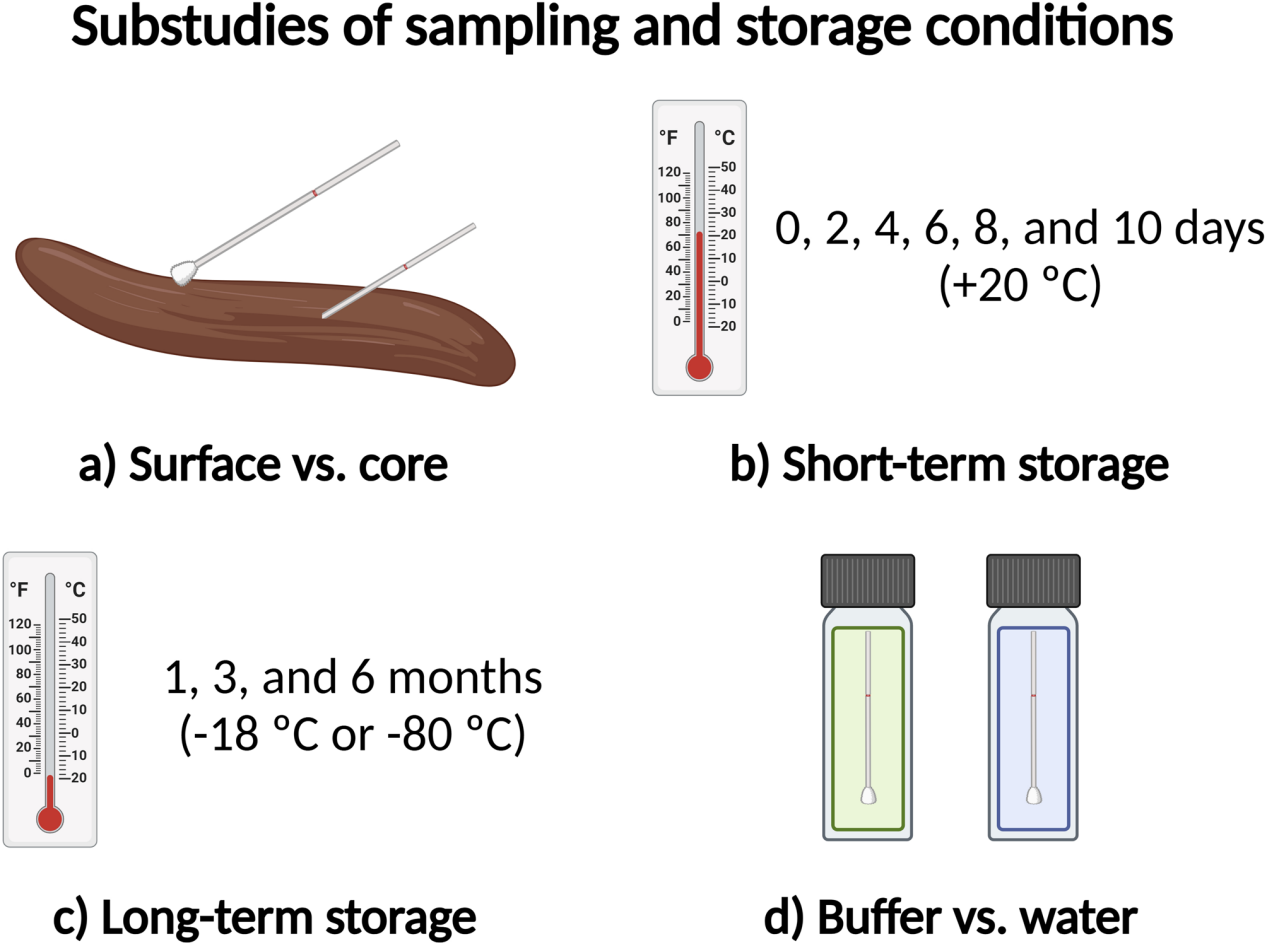
Overview of the four substudies. **(a)** Superficial sampling vs. sampling from the core of the stool. **(b)** Localized, pooled samples were stored at room temperature (+20 °C) until analysis. **(c)** Localized, pooled samples were stored in a freezer (–18 °C or −80 °C) until analysis. **(d)** Localized samples were stored in either transport medium or sterile water.

Furthermore, seven localized superficial samples were pooled and stored at either −18 °C or −80 °C for 1–6 months before DNA extraction to examine the effects of long-term storage. Finally, we investigated the impact of the transport medium on sample stability. The participants collected two localized samples, one of which was the transport medium that had been replaced with sterile water before collection. An overview of the four substudies is provided in Fig. 7.

### DNA extraction

DNA extraction from the fecal samples was performed via the AllPrep PowerFecal Pro DNA/RNA Kit (Cat. No. 80244; Qiagen, Hilden, Germany). Briefly, 200 µL of FIT sample buffer was transferred to a lysis tube provided by the manufacturer and eluted in 650 µL of buffer. Additionally, 25 µL of dithiothreitol was added. Both bead beating and chemical lysis were used in the DNA extraction process. DNA extraction was performed according to the manufacturer’s protocol. The concentrations of the extracted DNA were measured via a Qubit 1X dsDNA HS Assay Kit (Q33231, Thermo Fisher Scientific, Waltham, USA) and a Qubit™ 4 fluorometer (Q33226, Invitrogen, Carlsbad, USA).

### Polymerase chain reaction

After the DNA concentration in the extracts was quantified, polymerase chain reaction (PCR) was performed via the 16S Barcoding Kit SQK–RAB204 (Oxford Nanopore Technologies, Oxford, UK) according to the manufacturer’s instructions. The 16S primers 27F and 1492R were used to amplify the entire 16S region, including the nine hypervariable regions V1–V9, resulting in a PCR product of approximately 1,5 kBs. The primers also had rapid attachment chemistry and contained 24 barcodes to distinguish the samples during sequencing. For each PCR, 10 ng or 15 µL of extracted DNA was added, along with 10 µL of primer and 25 µL of LongAmp Taq 2X master mix (M0287L, New England Biolabs, Ipswich, USA). The 60-second initial denaturation at 95 °C was followed by 40 cycles of denaturation at 95 °C for 20 s, annealing at 55 °C for 30 s, extension at 65 °C for 120 s, and a final extension at 65 °C for 300 s.

The PCR products were purified via AMPure XP beads (A63881, Beckman Coulter, Indianapolis, USA) according to the 16S barcoding kit SQK–RAB204 protocol. In brief, the PCR products were transferred to a 1.5 mL centrifuge tube along with 30 µL AMPure XP beads. The samples were incubated on a Hula mixer (V.3A01, Life Technologies AS, Oslo, Norway). After 5 min, the samples were spun down and placed on a DynaMag™–2 (12321D, Invitrogen, Oslo, Norway) for 1 min until the beads had gathered on one side. The supernatant was discarded, and the beads were washed twice with 70% ethanol. After the second wash, the samples were spun down and placed on a magnet. The remaining liquid ethanol was pipetted away, and the samples were left to air-dry for 30 s to remove the last traces of ethanol. The samples were resuspended in 10 µL of 10 mM Tris–HCl (pH 8.0) with 50 mM NaCl and incubated at room temperature for 5 min. The samples were then placed back on the magnet and left for 1 min for the beads to gather on one side. Finally, the supernatant was transferred to a new 1.5 mL centrifuge tube, and the DNA concentration was measured via the Qubit 1X dsDNA HS Assay Kit, as described above. The amplification size was assessed during sequencing rather than by gel electrophoresis. Negative controls were used in the PCR.

### Library preparation and sequencing

Six purified samples were pooled in equal amounts to achieve a concentration of 5 fmol/µL, and rapid adapter protein (OXNTEXP–RAP001, Oxford Nanopore Technologies, Oxford, England) was added, along with sequencing buffer and loading beads, according to the manufacturer’s protocol. Each prepared library was loaded onto a new flow cell (version R9.4.1) and sequenced on a GridION Mk1 × 5 (ONT), with a sequencing process duration of 16 hours. We did not use quantitative microbiome profiling. The samples were sequenced in random order.

### Software and data processing

The MinKNOW software version 5.7.5 (ONT) performs base calling via Dorado version 7.1.4 in the “super accurate” setting. The threshold for the Phred score was set to 7, corresponding to a base call accuracy of greater than 80%. The full-length 16S rRNA sequences were demultiplexed using the EPI2ME™ (ONT) desktop agent fast 16S workflow. The nucleotide sequences were then identified using the BLAST from the United States National Library of Medicine, National Institutes of Health. We used the National Center for Biotechnology Information (NCBI) 16S + 18S rRNA database with default parameters (minimum length filter = 0, maximum length filter = 0, BLAST E-value filter = default [e = 0.01], minimum coverage % = 30, minimum identity % = 77, maximum target sequences = 3). Coverage information per read was calculated as the number of identical matches/query lengths. All read classifications were filtered for > 77% min. identity and > 30% coverage to remove spurious alignments, which are the default settings. Further analyses were performed using RStudio version 2023.09.1 (PBC, Boston, USA), running R version 4.3.1 (R Core Team). We analyzed the microbiome data using the phyloseq version 1.44.0 package^57^.

### Statistical calculations

Rarefaction curves were visualized for each sample to assess sequencing depth. To correct for differences in sequencing depth, we used normalization (i.e., the number of reads for each species divided by the total number of reads per sample) on all samples. We used Deming regression to fit a straight line to the data in the scatter plot, presented with the R^2^ value. Richness, a fundamental component of alpha diversity measures, was defined as the number of unique species in each sample. Alpha diversity was calculated via the Shannon diversity index. Differences in the alpha diversity between the two groups were calculated via the Wilcoxon rank-sum test. We used the Kruskal–Wallis test to determine the differences between three or more groups. Differences in beta diversity between the participants were calculated using the permutational analysis of variance (PERMANOVA) test. The relative abundances of the samples were visualized as stacked bar plots (detection = 0.5%; prevalence = 50%), and differences in proportions were calculated using the Wilcoxon rank-sum test, with the Benjamini‒Hochberg correction to adjust for the false discovery rate. We used the Canberra distance in principal coordinate analysis (PCoA). All values are presented as medians with interquartile ranges in parentheses. Adjusted P values < 0.05 were considered statistically significant.

## Electronic supplementary material

Below is the link to the electronic supplementary material.

Supplementary Material 1

## Data Availability

Owing to Danish legislation, sharing or storing raw biological information data outside approved Danish institutions is prohibited. You can contact the corresponding author if you have questions about the dataset.
