# Supplementary material for: Fecal microbiomes from screening sampling tubes are stable despite varying sampling and storage conditions
Source: Sci Rep. 2025 Jul 24;15:26951. doi: 10.1038/s41598-025-12506-5 (PMC12289992; doi:10.1038/s41598-025-12506-5)
Supplement: Supplementary file 1 — Supplementary Material 1 [file 41598_2025_12506_MOESM1_ESM.pdf]

Supplementary Material

Fecal microbiomes from screening sampling tubes are stable despite varying sampling and storage conditions

Anders Bech Jørgensen[1,¶,*], Louise Almer[2,¶], Birgitte Brandstrup[1,&], Lennart Friis-Hansen[3,&]

[1]Department of Surgery, Part of Copenhagen University Hospitals – Holbæk, Smedelundsgade 60, 4300 Holbæk, Denmark

[2]Center for Translational Research, Copenhagen University Hospital – Bispebjerg and Frederiksberg, Nielsine Nielsensvej 4B, 2400 Copenhagen NV, Denmark

[3]Department of Clinical Biochemistry, Copenhagen University Hospital – Bispebjerg and Frederiksberg, Nielsine Nielsensvej 4B, 2400 Copenhagen NV, Denmark

[¶]These authors contributed equally to this work

[&]These authors also contributed equally to this work

*Corresponding author

Anders Bech Jørgensen

E-mail: dr.bech@gmail.com

Supplementary Figures and Tables

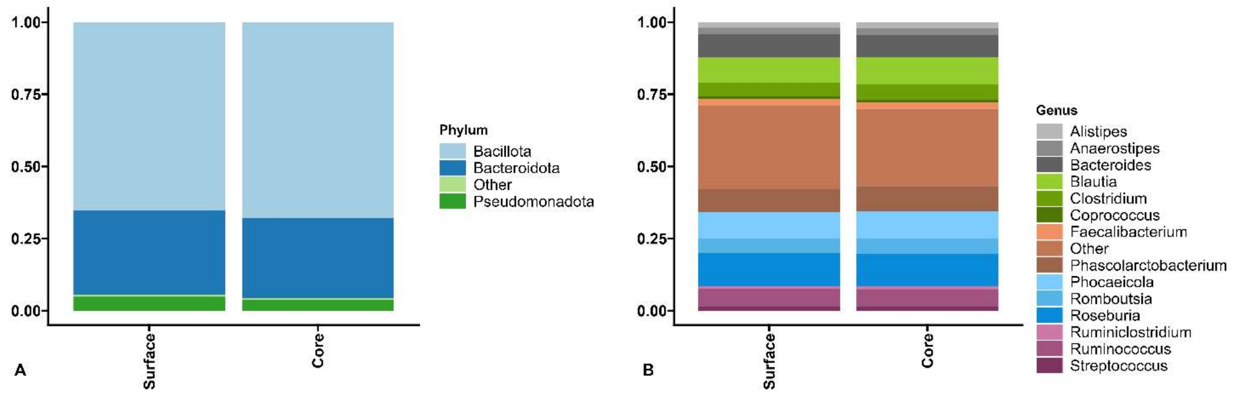

Supplementary Fig. S1. Relative abundance visualization, surface vs core samples.
A) Phylum level, B) Genus level.

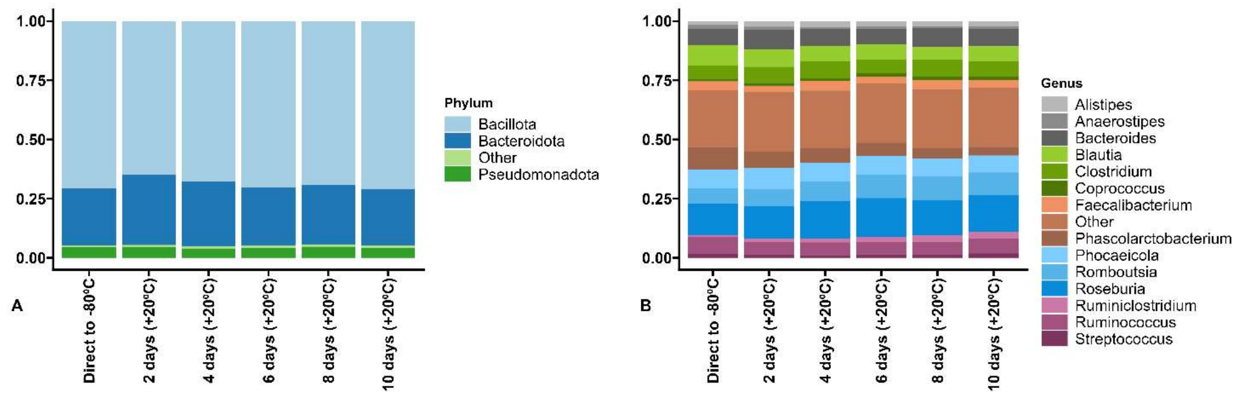

Supplementary Fig. S2. Relative abundance visualization, short-term storage (+20°C) for 2 to 10 days, compared with samples stored immediately at -80°C. A) Phylum level, B) Genus level.

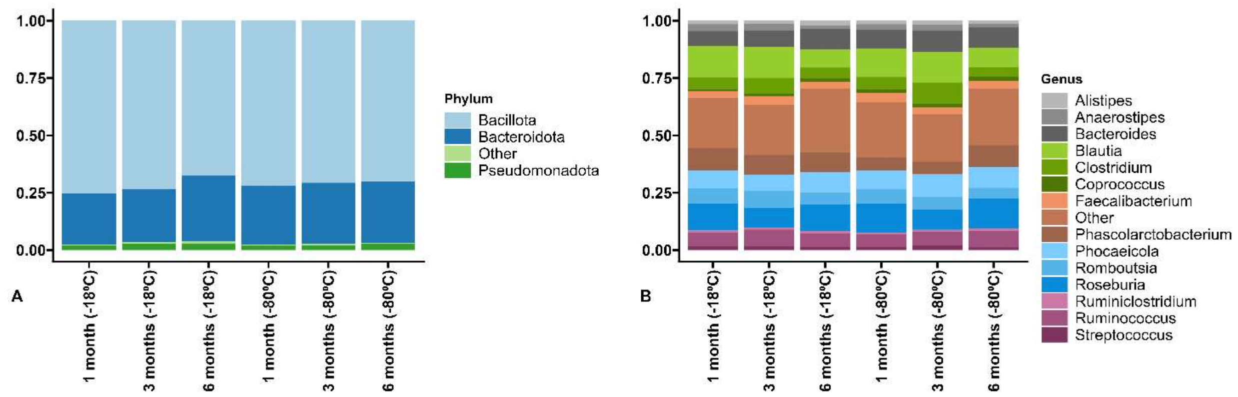

Supplementary Fig. S3. Relative abundance visualization, long-term storage (-18°C or -80°C) for 1, 3, or 6 months. A) Phylum level, B) Genus level.

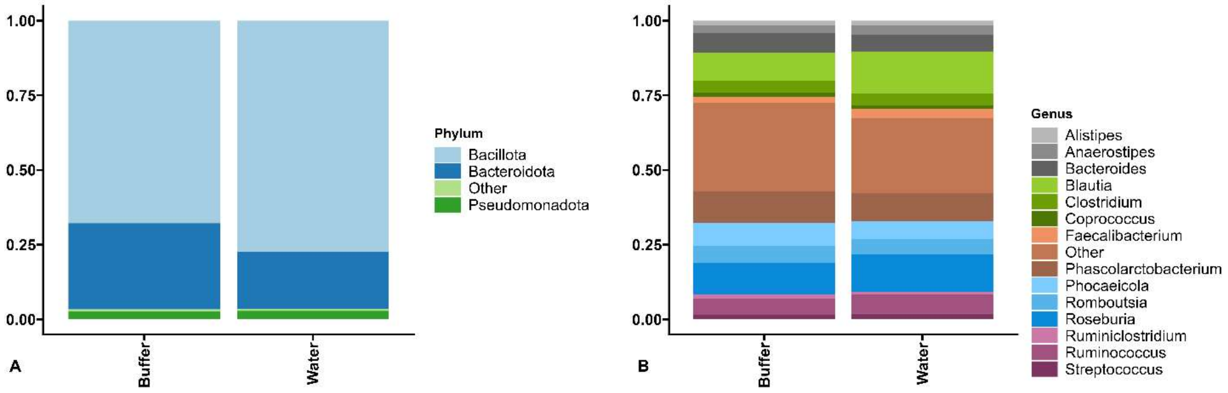

Supplementary Fig. S4. Relative abundance visualization, buffer medium vs water samples. A) Phylum level, B) Genus level.

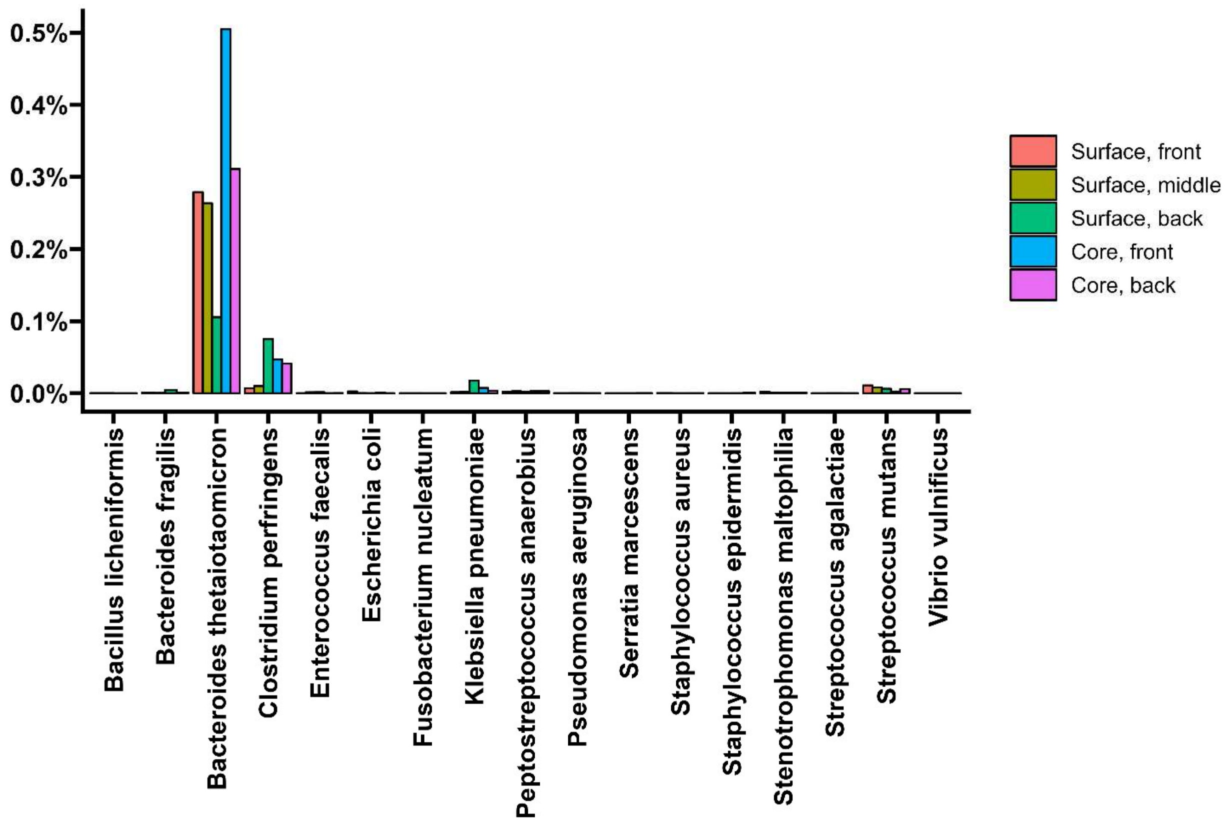

Supplementary Fig. S5. Prevalence of bacterial species that have the potential to produce the enzyme collagenase in surface vs. core samples.

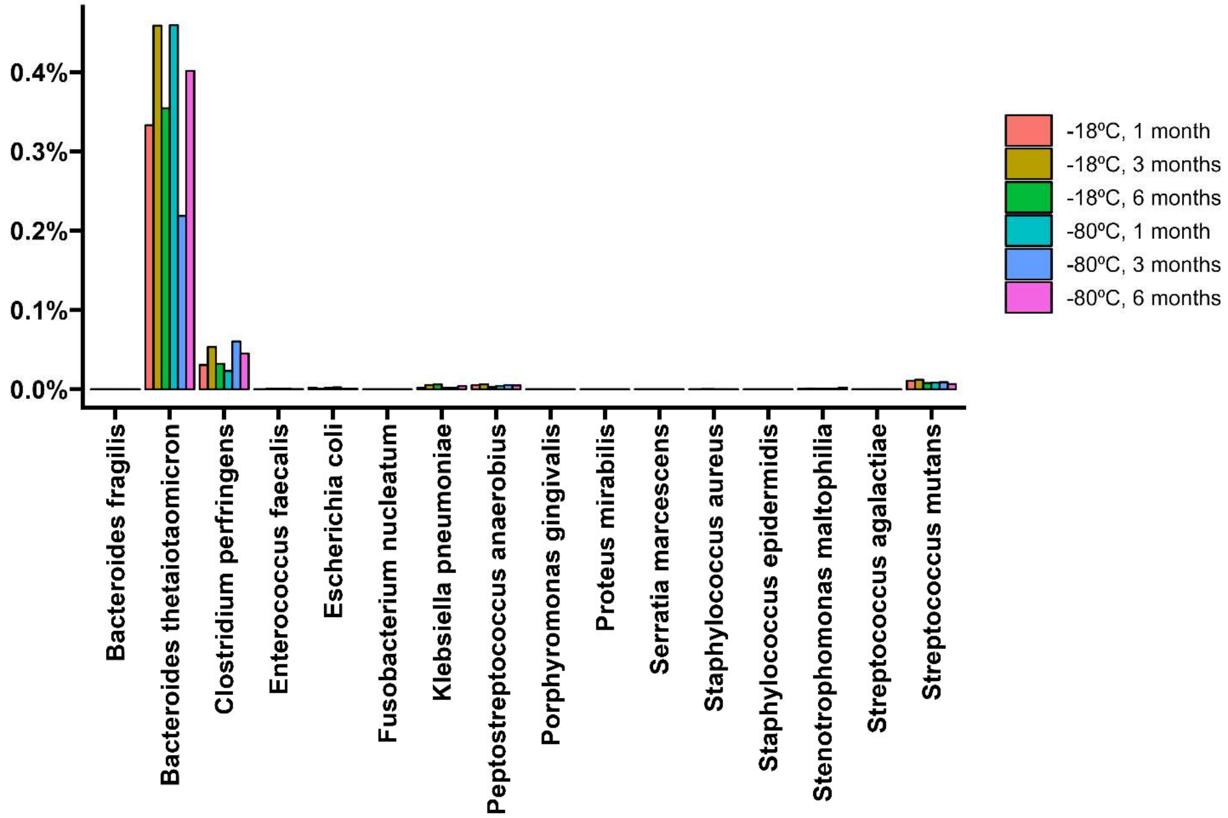

Supplementary Fig. S6. Prevalence of bacterial species that have the potential to produce the enzyme collagenase in long-term storage (-18°C or -80°C) samples.

	Richness	p-value	Shannon index	p-value
**Sampling site**		0.21		0.79
- surface	406 (335-591)		3.17 (2.97-3.46)	
- core	495 (416-645)		3.17 (2.98-3.34)	
**Storage**				
**Short-term (+20°C)**		0.97		0.95
- 0 days	480 (362-545)		3.10 (2.95-3.28)	
- 2 days	536 (361-575)		3.13 (3.01-3.50)	
- 4 days	437 (295-653)		3.10 (2.77-3.50)	
- 6 days	422 (332-614)		3.14 (2.69-3.66)	
- 8 days	425 (313-487)		3.22 (2.77-3.60)	
- 10 days	421 (351-483)		3.23 (2.81-3.67)	
**Long-term (-18°C)**		0.62		0.76
- 1 month	390 (348-496)		3.27 (2.96-3.47)	
- 3 months	456 (370-530)		3.20 (3.06-3.38)	
- 6 months	479 (400-576)		3.32 (3.11-3.54)	
**Long-term (-80°C)**		0.85		0.62
- 1 month	429 (369-524)		3.30 (2.92-3.48)	
- 3 months	485 (339-543)		3.25 (2.96-3.56)	
- 6 months	452 (301-518)		3.15 (2.95-3.42)	
**Transport medium**		0.46		0.96
- buffer	423 (345-614)		3.43 (2.88-3.53)	
- water	547 (408-665)		3.17 (2.99-3.41)	

Supplementary Table S1. Richness and alpha diversity (Shannon index) of all samples. Neither the Richness nor Shannon index has units. All values are medians with the interquartile range in parenthesis. P-values are calculated using the Kruskal-Wallis test.

STORMS checklist

The STORMS checklist. An editable version for adaptation and inclusion in publications is available from https://stormsmicrobiome.org

Number	Item	Recommendation	Item Source	Additional Guidance	Yes/No/NA	Comments or location in manuscript
**Abstract**						
1.0	Structured or Unstructured Abstract	Abstract should include information on background, methods, results, and conclusions in structured or unstructured format.	STORMS		Yes	Page 2, line 19-36
1.1	Study Design	State study design in abstract.	STORMS	See 3.0 for additional information on study design.	No	
1.2	Sequencing methods	State the strategy used for metagenomic classification.	STORMS	For example, targeted 16S by qPCR or sequencing, shotgun metagenomics, metatranscriptomics, etc.	Yes	Page 2, line 27-29
1.3	Specimens	Describe body site(s) studied.	STORMS		Yes	Page 2, line 25-26
**Introduction**						
2.0	Background and Rationale	Summarize the underlying background, scientific evidence, or theory driving the current hypothesis as well as the study objectives.	STORMS		Yes	Page 3-5, line 38-88
2.1	Hypotheses	State the pre-specified hypothesis. If the study is exploratory, state any pre-specified study objectives.	STORMS		Yes	Page 5, line 90-95

Methods						
3.0	Study Design	Describe the study design.	STORMS	Observational (Case-Control, Cohort, Cross-sectional survey, etc.) or Experimental (Randomized controlled trial, Non-randomized controlled trial, etc.). For a brief description of common study designs see: DOI: 10.11613/BM.2014.022<br><br>If applicable, describe any blinding (e.g. single or double-blinding) used in the course of the study.		Page 12, line 263
3.1	Participants	State what the population of interest is, and the method by which participants are sampled from that population. Include relevant information on physiological state of the subjects or stage in the life history of disease under study when participants were sampled.	STORMS	Examples of the population of interest could be: adults with no chronic health conditions, adults with type II diabetes, newborns, etc. This is the total population to whom the study is hoped to be generalizable to. The sampling method describes how potential participants were selected from that population.<br><br>If the participants are from a substudy of a larger study, provide a brief description of that study and cite that study.<br><br>Clearly state how cases and controls are defined.	Yes	Page 12, line 262-267

				An example of relevant physiological state might be pre/post menopausal for a vaginal microbiome study; examples of stage in the life history of disease could be whether specimens were collected during active or dormant disease, or before or after treatment.		
3.2	Geographic location	State the geographic region(s) where participants were sampled from.	MIxS: geographic location (country and/or sea,region)	Geographic coordinates can be reported to prevent potential ambiguities if necessary.	Yes – Denmark, as the study mimics the Danish Colorectal Cancer Screening	Page 12, line 263-264
3.3	Relevant Dates	State the start and end dates for recruitment, follow-up, and data collection.	STORMS	Recruitment is the period in which participants are recruited for the study. In longitudinal studies, follow-up is the date range in which participants are asked to complete a specific assessment. Finally, data collection is the total period in which data is being collected from participants including during initial recruitment through all follow-ups.	NA	This was not a longitudinal study

3.4	Eligibility criteria	List any criteria for inclusion and exclusion of recruited participants.	Modified STROBE	Among potential recruited participants, how were some chosen and others not? This could include criteria such as sex, diet, age, health status, or BMI.<br><br>If there is a primary and validation sample, describe inclusion/exclusion criteria for each.	Yes	Page 12, line 264-267
3.5	Antibiotics Usage	List what is known about antibiotics usage before or during sample collection.	STORMS	If participants were excluded due to current or recent antibiotics usage, state this here.<br><br>Other factors (e.g. proton pump inhibitors, probiotics, etc.) that may influence the microbiome should also be described as well.	Yes	Page 12, line 264-267
3.6	Analytic sample size	Explain how the final analytic sample size was calculated, including the number of cases and controls if relevant, and reasons for dropout at each stage of the study. This should include the number of individuals in whom microbiome sequencing was attempted and the number in whom microbiome sequencing was successful.	STORMS	Consider use of a flow diagram (see template at https://stormsmicrobiome.org/figures). Also state sample size in abstract.<br><br>If power analysis was used to calculate sample size, describe those calculations.	No	
3.7	Longitudinal Studies	For longitudinal studies, state how many follow-ups were conducted, describe sample size at follow-up by group or condition, and discuss any loss to follow-up.	STORMS	If there is loss to follow-up, discuss the likelihood that drop-out is associated with exposures, treatments, or outcomes of interest.	NA	This was not a longitudinal study

3.8	Matching	For matched studies, give matching criteria.	Modified STROBE	"Matched" refers to matching between comparable study participants as cases and controls or exposed / unexposed.<br><br>Indicate whether participants were individual or frequency matched and in what ratio were they matched (e.g. 1 case to 1 control).	NA	This was not a matched study
3.9	Ethics	State the name of the institutional review board that approved the study and protocols, protocol number and date of approval, and procedures for obtaining informed consent from participants.	STORMS		Yes	Page 12, line 259-260
4.0	Laboratory methods	State the laboratory/center where laboratory work was done.	STORMS	Provide a reference to complete lab protocols if previously published elsewhere such as on protocols.io. Note any modifications of lab protocols and the reason for protocol modifications.	Yes	Page 1 - affiliations
4.1	Specimen collection	State the body site(s) sampled from and how specimens were collected.	MIxS: sample collection device or method; host body site	Use terms from the Uber-anatomy Ontology (https://www.ebi.ac.uk/ols/ontologies/uberon) to describe body sites in a standardized format.	Yes	Page 13, line 284-289
4.2	Shipping	Describe how samples were stored and shipped to the laboratory.	STORMS	Include length of time from collection to receipt by the lab and if temperature control was used during shipping.	Yes	Page 13, line 278-282

4.3	Storage	Describe how the laboratory stored samples, including time between collection and storage and any preservation buffers or refrigeration used.	STORMS	State where each procedure or lot of samples was done if not all in the same place.<br><br>Include reagent/lot/catalogue #s for storage buffers.	Yes	Page 13, line 278-282
4.4	DNA extraction	Provide DNA extraction method, including kit and version if relevant.	MIxS: nucleic acid extraction	If any DNA quantification methods were used prior to DNA amplification or at the pooling step of library preparation, state so here.	Yes	Page 13-14, line 289-297
4.5	Human DNA sequence depletion or microbial DNA enrichment	Describe whether human DNA sequence depletion or enrichment of microbial or viral DNA was performed.	STORMS		No	
4.6	Primer selection	Provide primer selection and DNA amplification methods as well as variable region sequenced (if applicable).	MIxS: pcr primers		Yes	Page 14, line 311-313
4.7	Positive Controls	Describe any positive controls (mock communities) if used.	STORMS	If used, should be deposited under guidance provided in the 8.X items.	No	
4.8	Negative Controls	Describe any negative controls if used.	STORMS	If used, should be deposited under guidance provided in the 8.X items.	Yes, in PCR	Page 15, line 334-335
4.9	Contaminant mitigation and identification	Provide any laboratory or computational methods used to control for or identify microbiome contamination from the environment, reagents, or laboratory.	STORMS	Includes filtering of reagents and other steps to minimize contamination. It is relevant to state whether the specimens of interest have low microbial load, which makes contamination especially relevant.	No	

4.10	Replication	Describe any biological or technical replicates included in the sequencing, including which steps were replicated between them.	STORMS	Replication may be biological (redundant biological specimens) or technical (aliquots taken at different stages of analysis) and used in extraction, sequencing, preprocessing, and/or data analysis.	No	
4.11	Sequencing strategy	Major divisions of strategy, such as shotgun or amplicon sequencing.	MIxS: sequencing method	For amplicon sequencing (for example, 16S variable region), state the region selected. State the model of sequencer used.	Yes	Page 16, line 337-344
4.12	Sequencing methods	State whether experimental quantification was used (QMP/cell count based, spike-in based) or whether relative abundance methods were applied.	STORMS	These include read length, sequencing depth per sample (average and minimum), whether reads are paired, and other parameters.	Yes, read length	Page 16, line 337-344
4.13	Batch effects	Detail any blocking or randomization used in study design to avoid confounding of batches with exposures or outcomes. Discuss any likely sources of batch effects, if known.	STORMS	Sources of batch effects include sample collection, storage, library preparation, and sequencing and are commonly unavoidable in all but the smallest of studies.	Yes	Page 16, line 343-344
4.14	Metatranscriptomics	Detail whether any mRNA enrichment was performed and whether/how retrotranscription was performed prior to sequencing. Provide size range of isolated transcripts. Describe whether the sequencing library was stranded or not. Provide details on sequencing methods and platforms.	STORMS	Provide details on any internal standards which may have been used as well as parameters and versions of any software or databases used.	NA	
4.15	Metaproteomics	Detail which protease was used for digestion. Provide details on proteomic methods and platforms (e.g. LC-MS/MS, instrument type, column type, mass range, resolution, scan speed, maximum	STORMS	Provide details on any internal standards which may have been used as well as parameters and versions of any software or databases used.	NA	

		injection time, isolation window, normalised collision energy, and resolution).				
4.16	Metabolomics	Specify the analytic method used (such as nuclear magnetic resonance spectroscopy or mass spectrometry). For mass spectrometry, detail which fractions were obtained (polar and/or non-polar) and how these were analyzed. Provide details on metabolomics methods and platforms (e.g. derivatization, instrument type, injection type, column type and instrument settings).	STORMS	Provide details on any internal standards which may have been used as well as parameters and versions of any software or databases used.	NA	
5.0	Data sources/ measurement	For each non-microbiome variable, including the health condition, intervention, or other variable of interest, state how it was defined, how it was measured or collected, and any transformations applied to the variable prior to analysis.	MIxS: host disease status	State any sources of potential bias in measurements, for example multiple interviewers or measurement instruments, and whether these potential biases were assessed or accounted for in study design.<br><br>Use terms from a standardized ontology such as the Experimental Factor Ontology (https://www.ebi.ac.uk/efo/) to describe variables of interest in a standardized format.	NA	

6.0	Research design for causal inference	Discuss any potential for confounding by variables that may influence both the outcome and exposure of interest. State any variables controlled for and the rationale for controlling for them.	STORMS	For causal inference, this item refers to describing the assumptions that would be required to draw causal inferences from observational data. See Vujkovic-Cvijin, I., Sklar, J., Jiang, L. et al. Host variables confound gut microbiota studies of human disease. Nature 587, 448–454 (2020). https://doi.org/10.1038/s41586-020-2881-9 for more details on confounding in observational microbiome studies.<br><br>For example, hypothesized confounders may be controlled for by multivariable adjustment. Consider using a directed acyclic graph (DAG) to describe your causal model and justify any variables controlled for. DAGs can be made using www.dagitty.net.	NA	
6.1	Selection bias	Discuss potential for selection or survival bias.	STORMS	Selection bias can occur when some members of the target study population are more likely to be included in the study/final analytic sample than others. Some examples include survival bias (where part of the target study population is more likely to die before they can be studied), convenience sampling (where members of the target study population are not selected at random), and loss to follow-up (when	NA	

				probability of dropping out is related to one of the things being studied).		
7.0	Bioinformatic and Statistical Methods	Describe any transformations to quantitative variables used in analyses (e.g. use of percentages instead of counts, normalization, rarefaction, categorization).	STORMS	If a variable is analyzed using different transformations, state rationale for the transformation and for each analyses which version of the variable is used.<br><br>In case of any complex or multistep transformations, give enumerated instructions for reproducing those transformations.	Yes	Page 17, line 364-366
7.1	Quality Control	Describe any methods to identify or filter low quality reads or samples.	MIxS: sequence quality check	If samples were excluded based on quality or read depth, list the criteria used, the number of samples excluded, and the final sample size after quality control.	Yes, rarefaction curves	Page 16-17, line 346-361
7.2	Sequence analysis	Describe any taxonomic, functional profiling, or other sequence analysis performed.	MIxS: feature prediction; similarity search method		Yes	Page 17, line 363-376

7.3	Statistical methods	Describe all statistical methods.	Modified STROBE	Describe any statistical tests used, exploratory data analysis performed, dimension reduction methods/unsupervised analysis, alpha/beta metrics, and/or methods for adjusting for measurement bias.<br><br>If multiple statistical methods are possible, discuss why the methods used were selected.<br><br>If a multiple hypothesis testing correction method was used, describe the type of correction used.<br><br>State which taxonomic levels are analyzed.	Yes	Page 17, line 363-376
7.4	Longitudinal analysis	If the study is longitudinal, include a section that explicitly states what analysis methods were used (if any) to account for grouping of measurements by individual or patterns over time.	STORMS		NA	
7.5	Subgroup analysis	Describe any methods used to examine subgroups and interactions.	STROBE		NA	
7.6	Missing data	Explain how missing data were addressed.	STROBE	"Missing data" refers to participant measurements such as covariates, exposures, outcomes, or time points that should have been collected but were not, not to zeros in taxonomic abundance tables or data points not applicable to that observation.	Yes	Page 5, page 104-105

7.7	Sensitivity analyses	Describe any sensitivity analyses.	STROBE		No	
7.8	Findings	State criteria used to select findings for reporting.	STORMS	For example, false discovery rate with total number of tests, effect size threshold, significance threshold, microbes of interest.	No	
7.9	Software	Cite all software (including read mapping software) and databases (including any used for taxonomic reference or annotating amplicons, if applicable) used. Include version numbers.	Modified STREGA	Installed packages, add-ons or libraries should be stated and cited in addition to the software used.<br><br>All parameters employed that differ from the default of that software/version should be provided.<br><br>This is in addition to, not a replacement for, publishing of code as outlined in the section Reproducible Research.	Yes	Page 17, line 359-361
8.0	Reproducible research	Make a statement about whether and how others can reproduce the reported analysis.	STORMS	Any protected information that has been excluded or provided under controlled access should be listed along with any relevant data access procedures. "On request from authors" is not sufficiently detailed; formal data access procedures and conditions should be defined.<br><br>If data are unavailable, state so clearly.	No – data sharing not allowed according to Danish legislation	

				Consider using a specialized rubric for reproducible research (such as: https://mbio.asm.org/content/9/3/e00525-18.short).<br><br>Consider preregistering the study protocol (such as on osf.io or https://plos.org/open-science/preregistration/).		
8.1	Raw data access	State where raw data may be accessed including demultiplexing information.	STORMS	Robust, long-term databases such as those hosted by NCBI and EBI are preferred. If using a private repository, provide rationale.	No – data sharing not allowed according to Danish legislation	
8.2	Processed data access	State where processed data may be accessed.	STORMS	Unfiltered data should be provided.<br><br>Robust, long-term databases such as those hosted by NCBI and EBI-EMBL are preferred. Repositories like zenodo (https://zenodo.org/) or publisso (https://www.publisso.de/en/working-for-you/doi-service/)<br>can be used to provide a DOI and long-term storage for processed datasets, even those which cannot be published openly.	No – data sharing not allowed according to Danish legislation	

8.3	Participant data access	State where individual participant data such as demographics and other covariates may be accessed, and how they can be matched to the microbiome data.	STORMS	If re-categorized, transformed, or otherwise derived variables were used in the analysis, these variables or code for deriving them should be provided.<br><br>Examples of how participant data can be matched to microbiome data are: using the same set of anonymized identifiers, or using different anonymized identifiers but providing a map.<br><br>Provided data should be sufficient to independently replicate the current analysis.	No – data sharing not allowed according to Danish legislation	
8.4	Source code access	State where code may be accessed.	STORMS	If a standard or formalized workflow was employed, reference it here.	NA – does not make sense as data is not available	
8.5	Full results	Provide full results of all analyses, in computer-readable format, in supplementary materials.	STORMS	For example, any fold-changes, p-values, or FDR values calculated, provided as a spreadsheet.<br><br>Use a machine-readable, plain-text format such as csv or tsv.	Yes	Supplementary Material

Results

9.0	Descriptive data	Give characteristics of study participants (e.g. dietary, demographic, clinical, social) and information on exposures and potential confounders.	STROBE	Typically reported in a table included in the paper or as a supplementary table. Indicate number of participants with missing data for each variable of interest.<br><br>This includes environmental and lifestyle factors that may affect the relationship between the microbiome and the condition of interest. Participant diet and medication use should be summarized, if known.<br><br>At minimum, age and sex of all participants should be summarized.	Yes	Page 5, line 100-101
10.0	Microbiome data	Report descriptive findings for microbiome analyses with all applicable outcomes and covariates.	STORMS	This includes measures of diversity as well as relative abundances. These descriptive findings should be reported both for the sample overall and for individual groups.	Yes	Page 5-8, line 103-159
10.1	Taxonomy	Identify taxonomy using standardized taxon classifications that are sufficient to uniquely identify taxa.	STORMS	If not using full taxonomic hierarchy, make sure it is clear whether names stated are species, genera, family, etc.<br><br>Italicize genus/species pairs. Consult journal guidelines or standardized references on taxonomic nomenclature. For instance, https://wwwnc.cdc.gov/eid/page/scientific-nomenclature	Yes	Page 5-8, line 103-159

10.2	Differential abundance	Report results of differential abundance analysis by the variable of interest and (if applicable) by time, clearly indicating the direction of change and total number of taxa tested.	STORMS	If there are more than two groups, include omnibus (multigroup) test results if applicable to the research question.<br><br>If applicable, reported effect sizes should include a measure of uncertainty such as the confidence interval.	Yes	Page 5-8, line 103-159
10.3	Other data types	Report other data analyzed--e.g. metabolic function, functional potential, MAG assembly, and RNAseq.	STORMS		NA	
10.4	Other statistical analysis	Report any statistical data analysis not covered above.	STORMS	This could include subgroup analysis, sensitivity analyses, and cluster analysis.<br><br>Visualizations should be easily interpretable and colorblind-friendly. The caption and/or main text should provide a detailed description of visualizations for visually-impaired readers.	NA	
**Discussion**						
11.0	Key results	Summarise key results with reference to study objectives	STROBE		Yes	Page 8, line 164-172

12.0	Interpretation	Give a cautious overall interpretation of results considering objectives, limitations, multiplicity of analyses, results from similar studies, and other relevant evidence.	STROBE	Define or clarify any subjective terms such as "dominant," "dysbiosis," and similar words used in interpretation of results.<br><br>When interpreting the findings, consider how the interpretation of the findings may be summarized or quoted for the general public such as in press releases or news articles.<br><br>If causal language is used in the interpretation (such as "alters," "affects," "results in," "causes," or "impacts"), assumptions made for causal inference should be explicitly stated as part of 6.0 and 13.0.<br><br>Distinguish between function potential (ie inferred from metagenomics) and observed activity (ie metatranscriptomic, metabolomic, proteomic) if discussing microbial function.	Yes	Page 8-11, line 174-228
13.0	Limitations	Discuss limitations of the study, taking into account sources of potential bias or imprecision.	STROBE	Also consider limitations resulting from the methods (especially novel methods), the study design, and the sample size.	Yes	Page 11, line 232-240
13.1	Bias	Discuss any potential for bias to influence study findings.	STORMS	May include sampling method, representativeness of study	Yes	Page 11, line 232-240

				participants, or potential confounding.		
13.2	Generalizability	Discuss the generalisability (external validity) of the study results	STROBE	To what populations or other settings do you expect the conclusions to generalize?	Yes	Page 11, line 237-239
14.0	Ongoing/future work	Describe potential future research or ongoing research based on the study's findings.	STORMS		Yes	Page 11, line 237-240
**Other information**						
15.0	Funding	Give the source of funding and the role of the funders for the present study and, if applicable, for the original study on which the present article is based	STROBE		Yes	Page 25, line 560-564
15.1	Acknowledgements	Include acknowledgements of those who contributed to the research but did not meet critera for authorship.	STORMS	For general guidelines on authorship, see http://www.icmje.org and https://www.elsevier.com/authors/journal-authors/policies-and-ethics/credit-author-statement	Yes	Page 24, line 530-531
15.2	Conflicts of Interest	Include a conflicts of interest statement.	STORMS		Yes	Page 25, line 557-558
16.0	Supplements	Indicate where supplements may be accessed and what materials they contain.	STORMS		Yes	Noted in manuscript (Supplementary Figures and Tables)

17.0	Supplementary data	Provide supplementary data files of results with for all taxa and all outcome variables analyzed. Indicate the taxonomic level of all taxa.	STORMS	Depending on the analysis performed, examples of the supplemental results included could be mean relative abundance, differential abundance, raw p-value, multiple hypothesis testing-adjusted p-values, and standard error.<br><br>All discussed taxa should include the taxonomic level (e.g. class, order, genus).	Yes	Supplementary data are presented in this document
